# Myostatin Mutation Enhances Bovine Myogenic Differentiation through PI3K/AKT/mTOR Signalling via Removing DNA Methylation of RACK1

**DOI:** 10.3390/cells12010059

**Published:** 2022-12-23

**Authors:** Yiping Zhao, Xiaoxia Xia, Qiaomeng Wang, Debao Hu, Linlin Zhang, Xin Li, Xiangbin Ding, Hong Guo, Yiwen Guo

**Affiliations:** College of Animal Science and Veterinary Medicine, Tianjin Agricultural University, Tianjin 300384, China

**Keywords:** myostatin, DNA methylation, RACK1, PI3K/AKT/mTOR signaling, myogenic differentiation

## Abstract

Myostatin (MSTN) is a negative regulator of skeletal muscle development and plays an important role in muscle development. Fluctuations in gene expression influenced by DNA methylation are critical for homeostatic responses in muscle. However, little is known about the mechanisms underlying this fluctuation regulation and myogenic differentiation of skeletal muscle. Here we report a genome-wide analysis of DNA methylation dynamics in bovine skeletal muscle myogenesis after myostatin editing. We show that, after myostatin editing, an increase in TETs (DNA demethylases) and a concomitant increase in the receptor for activated C kinase 1 (RACK1) control the myogenic development of skeletal muscle. Interestingly, enhancement of PI3K/AKT/mTOR signaling by RACK1 appears to be an essential driver of myogenic differentiation, as it was associated with an increase in myogenic differentiation marker factors (MyHC and MyoG) during muscle differentiation. Overall, our results suggest that loss of myostatin promotes the myogenic differentiation response in skeletal muscle by decreasing DNA methylation of RACK1.

## 1. Introduction

Skeletal muscle is a vital tissue in the body. Loss of its functional or regenerative properties leads to debilitating musculoskeletal diseases. Myostatin is a key factor that negatively regulates muscle development. It is important to treat muscle atrophy and metabolic disorders by targeting the myostatin signaling pathway [1].

DNA methylation is a well-studied process. DNA de novo methyltransferases (DMNT3A and DMNT3B) are responsible for establishing methylation patterns and DNA maintenance methyltransferases (DMNT1) are responsible for maintaining methylation patterns [2,3]. The DNA methylation pattern in the genome is not static, which stems from the dynamic transition of DNA methylation and demethylation during the growth and metabolism of the organism. Previously, cytosine methylation was considered a very stable modification, so demethylation could only occur passively, i.e., in the absence of DNMT1 remethylation after replication of methylated DNA, via progressive dilution, occurs. Currently, many studies have identified another mode of regulation in mammals: the oxidation of 5mC transduced by Ten-11 translocation (TET) methylcytosine dioxygenases (TET1, TET2, TET3) to generate 5-hydroxymethyl cytosine (5hmC), 5-formylcytosine (5fC) and 5-carboxycytosine (5caC), and finally 5hmC is actively reverted to unmodified C [4,5,6,7]. The active demethylation pattern of DNA depends mainly on the TET enzyme family TET1, TET2, and TET3. The TET enzymes generate and protect hypomethylation in key regulatory regions throughout the genome [8,9]. Structurally, while all TET family members contain a conserved C-terminal cysteine-rich catalytic domain (CD), only TET1 and TET3 possess the N-terminal Cys-Xaa-Xaa-Cys (CXXC) domain, which is a potential DNA-binding module with two CXXXXXC repeats [10,11]. In contrast to TET1 and TET3, TET2 lost the CXXC zinc finger domain involved in binding unmethylated CpG sequences during evolution [12]. The expression of TET1, TET2, and TET3 differs in early embryonic development. Only TET3 is highly expressed in mouse oocytes and zygotes and is responsible for the hydroxylation of 5mC in the paternal pronucleus of late prokaryotic zygotes, whereas TET1 and TET2 are strongly expressed before embryo implantation [13,14,15].

We find that the DNA demethylation-RACK1-PI3K/AKT/mTOR axis may be a key factor in the influence of myostatin on muscle development. We used genome-wide DNA methylation sequencing (WGBS) to investigate the relationship between myostatin deletion-induced skeletal muscle enlargement and DNA methylation. We identified RACK1 as a direct target that regulates dynamic changes in DNA methylation in bovine skeletal muscle, with the enzyme TET1 playing an important role. We further investigated the role of demethylases and RACK1 in regulating muscle homeostasis using a cellular model with exogenous expression of RACK1 and inhibition of the DNA demethylation pathway. In addition, we found that RACK1 may be one of the activators of the PI3K/AKT/mTOR pathway. Taken together, our results describe a possible mechanism by which DNA methylation induced by myostatin depletion regulates skeletal myogenic differentiation in cattle and provide new insights into the relationship between DNA methylation and muscle development.

## 2. Methods

### 2.1. Animal and Muscle Tissue Collection

The experimental ranch of Inner Mongolia University provided Luxi yellow cattle muscle samples. The experimental group and the control group were clinically healthy *MSTN^+/−^* Luxi cattle (group name: ZJY, serial numbers: Z61023, Z61020, Z61004, Z61128, and Z61117) and wild-type Luxi yellow cattle (group name: FZ., serial numbers: F81209, F81210, F81208, F81214, and F81199), each with five heads. The muscle samples were in vivo samples from the buttock muscle of the cattle, taken with a sampling gun (Angiotech, USA). The tissues were immediately frozen in liquid nitrogen (Junliangcheng Changfu Gas Co., Ltd., Tianjin, China) for WGBS-seq, RNA-seq, and pyrosequencing. All the experiments were conducted in strict accordance with the recommendations in the guidelines for Animal Protection and Utilization of Inner Mongolia University and approved by the Animal Welfare Committee of Inner Mongolia University.

### 2.2. WGBS Library Preparation, Sequencing, Quality Analysis, and Mapping

The detailed methods were described in Supplementary Material File S1.

### 2.3. Identification of Differentially Methylated Regions (DMRs) and the GO Enrichment Analyses of WGBS

The detailed methods are described in Supplementary Material File S2.

### 2.4. Pyrosequencing Assay

The detailed methods are described in Supplementary Material File S3.

### 2.5. Bovine Skeletal Muscle Satellite Cells Resuscitation and Induction of Differentiation Culture, Cell Transfection Assay, and Immunofluorescence Staining

The detailed methods are described in Supplementary Material File S4.

### 2.6. Extraction of Total Cellular RNA, Synthesis of the First Strand of cDNA and qRT-PCR Assay

The detailed methods are described in Supplementary Material File S5.

### 2.7. Western Blot Assay and CCK-8 Analysis

The detailed methods are described in Supplementary Material File S6.

### 2.8. Ch-IP Experiments and Co-IP Experiments

The detailed methods are described in Supplementary Material File S7.

### 2.9. Hydroxymethylcytosine (5hmC) Hydroxylase TET Activity Assay (ELISA Assay)

The detailed methods are described in Supplementary Material File S8.

### 2.10. Construction of Overexpression Vector and Synthesis of siRNA

The detailed methods are described in Supplementary Material File S9.

### 2.11. Bioinformatics Analysis

The UniProt website (http://www.uniprot.org/ accessed on 1 April 2020) was used to find protein function [16]. DAVID Bioinformatics Resources 6.8 (https://david.ncifcrf.gov/ accessed on 1 April 2020) was used to perform the GO annotation and KEGG pathway analysis of differentially expressed genes [17]. All interactions and construct networks were performed in the STRING 11.0 database (http://string-db.org/ accessed on 1 April 2020) [18].

### 2.12. Statistical Analysis

All results are presented as mean ± SEM based on three independent experiments. The qRT-PCR results used TUBB as an internal reference, and the relative gene expression levels were obtained by the 2^−∆∆Ct^ method. The Western blot results were quantified by Image Lab software, and β-Tubulin was used as an internal reference for Western blot results. Differences between groups were statistically analyzed using Student’s *t*-test. *p* < 0.05 was considered statistically significant (* *p* < 0.05 and ** *p* < 0.01 and N.S. *p* > 0.05).

## 3. Results

### 3.1. Genome-Wide DNA Methylation Profiling and Data Accuracy Validation

**Figure 1:**
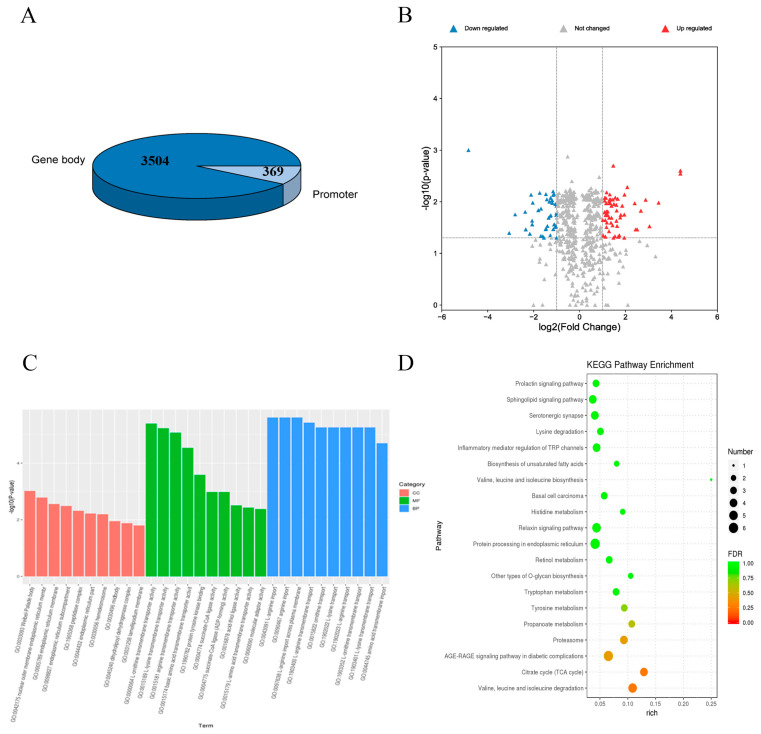
Statistics of DMGs. (**A**) Statistics of total DMRs identified by WGBS-seq. (**B**) Volcano diagram of DMGs in promoter regions. (**C**) GO promoter region analysis. (**D**) KEGG bubble chart of promoter region.

A global DNA methylation analysis of *MSTN^+/−^*-edited bovine muscle (Z) and wild-type bovine muscle (F) was carried out using WGBS. Details of sequencing data quality and genome-wide DNA methylation analysis are provided in Table 1 and Supplementary Materials File S11. We identified 3873 differentially methylated regions (DMRs) in WGBS, including 3504 in the gene body region and 369 in the promoter region, and generated volcano maps for differentially methylated genes (DMGs) in the promoter region (Figure 1A,B). To explore the critical genes in skeletal muscle development, we set two constraints for association analysis: first, we identified the 39 DMGs in the promoter region, fold change ≥ 2.00 or ≤0.50, and *p* ≤ 0.05. Second, we used RNA-seq data [19] from the same sample to search for overlapping genes whose methylation level is inversely correlated with transcription level. We identified 4 upregulated DMGs and 11 downregulated DMGs in the promoter region. We identified them as node genes in interaction networks associated with muscle development pathways. We annotated DMGs discovered in DMRs, using the GO and KEGG databases. Promoter regions are based on the GO database. Terms that play a critical role in muscle growth and are significantly enriched (corrected *p*  <  0.05) include skeletal muscle hypertrophy, negative regulation of skeletal muscle hypertrophy, and regulation of skeletal muscle adaptation (Figure 1C). We show 30 KEGG terms related to muscle development with DMG-adjusted *p* values <  0.05 (Figure 1D). According to the results, these DMGs may affect muscle development.

**Figure 2:**
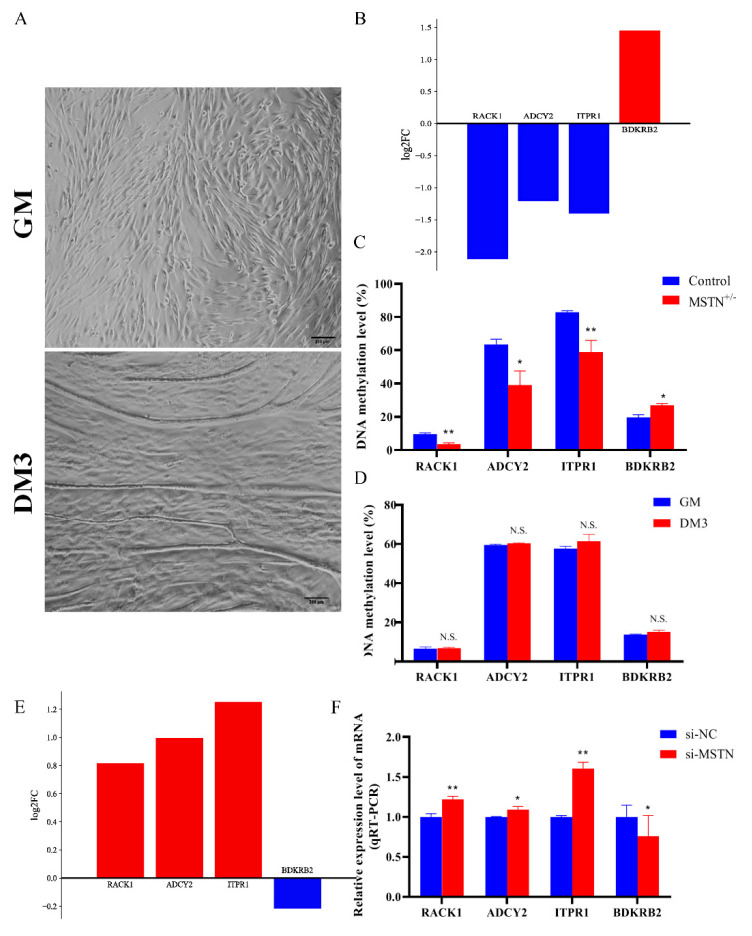
Accuracy verification of WGBS data. (**A**) Cell maps of GM and DM3 (100×). (**B**) Upregulation or downregulation of RACK1, ADCY2, ITPR1, and BDKRB2 in WGBS-seq data. (**C**) Histogram of DNA methylation levels (pyrosequencing) of muscle samples. (**D**) Histogram of DNA methylation levels (pyrosequencing) of cell samples. (**E**) Upregulation or downregulation of RACK1, ADCY2, ITPR1, and BDKRB2 in RNA-seq data. (**F**) RT-PCR detection of RACK1, ADCY2, ITPR1, and BDKRB2 transcript levels. Differences between groups were statistically analyzed using Student’s *t*-test. *p* < 0.05 was considered statistically significant (* *p* < 0.05 and ** *p* < 0.01). And N.S., not significant (*p* > 0.05).

We randomly selected four genes from the screened dataset for pyrosequencing to check the accuracy of the WGBS-seq data. Their DMR details can be found in Supplementary Material File S10. The cells in the proliferative phase (GM) and on the third day of the differentiation phase (DM3) for pyrosequencing were in good condition (Figure 2A). Pyrosequencing showed that the methylation levels of RACK1 (*p* < 0.01), ITPR1 (*p* < 0.01), and ADCY2 (*p* < 0.05) decreased and the methylation level of BDKRB2 increased (*p* < 0.05), consistent with the WGBS-seq data (Figure 2B,C). Interestingly, the DNA methylation levels of these DMGs did not change significantly in the sequencing results of the cell samples before and after normal cell differentiation (*p* > 0.05) (Figure 2D), suggesting that the changes in DNA methylation levels of these genes may be due to downregulation of MSTN gene expression. We compared the RNA-seq data with the results from RT-PCR and found that deletion of myostatin resulted in an increase in RACK1 (*p* < 0.01), ITPR1 (*p* < 0.01), and ADCY2 (*p* < 0.05) transcript levels and a decrease in BDKRB2 transcript levels (Figure 2E,F), consistent with the RNA-seq results.

### 3.2. Global DNA Demethylation Patterns Resulting from Myostatin Deletion

**Figure 3:**
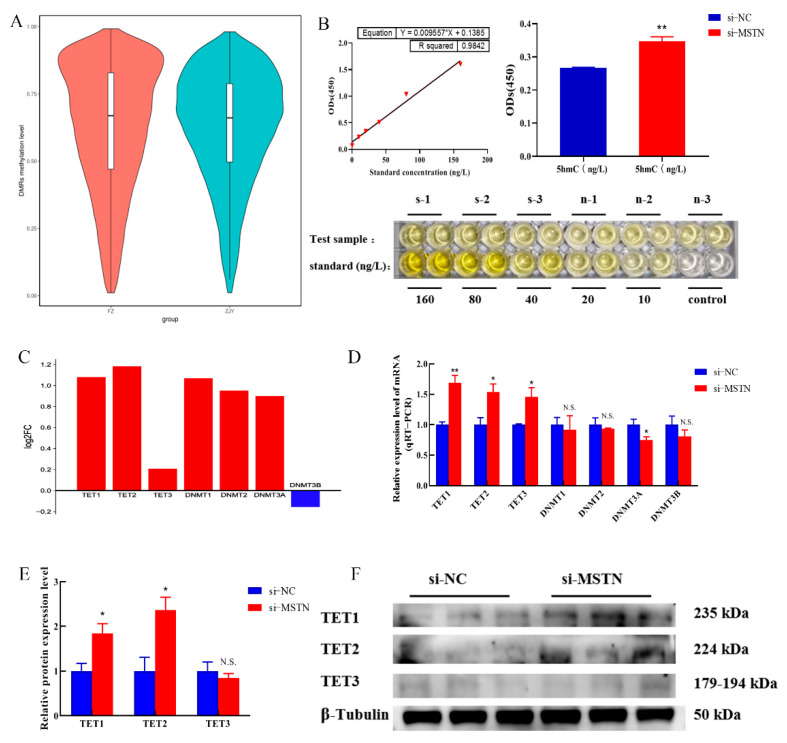
DNA methylation pattern resulting from myostatin editing. (**A**) Violin diagram of WGBS-seq data. (**B**) Hydroxymethylcytosine (5hmC) hydroxylase TET activity assay. Standardized fitted curve of 5hmc activity assay (**top left**), quantitative results of 5hmc activity assay (**top right**), and ELISA assay well plot (**bottom**). (**C**) Histogram of upregulation or downregulation of DNMT and TET family genes in RNA-seq. (**D**) qRT-PCR detection of transcription level of DNMT and TET family genes. (**E**) Quantitative analysis results of protein gray value. (**F**) Representative bands of the TET family. Differences between groups were statistically analyzed using Student’s *t*-test. *p* < 0.05 was considered statistically significant (* *p* < 0.05 and ** *p* < 0.01). And N.S., not significant (*p* > 0.05).

The WGBS-seq results showed that the rank of DNA methylation of MSTN-edited (ZJY) DMRs was lower than that of the wild type (FZ) (Figure 3A). The results of the detection of 5-hydroxymethylcytosine (5hmC) hydroxylase TET activity showed that the upregulation of 5hmC hydroxylase TET activity in cells was highly significant (*p* < 0.01) (Figure 3B). This indicates that myostatin gene deletion reduced the global DNA methylation levels of cells and dominated DNA demethylation modifications.

Subsequently, myostatin damage resulted in higher fold differences in TET1 and TET2 than in TET3 and DNMT family genes (Figure 3C). qPCR showed increased transcription in TET1, TET2, and TET3 (*p* < 0.05) and no significant changes in other genes (*p* > 0.05) except downregulation of DNMT3A levels (Figure 3D). Western blotting showed increased protein levels of TET1 and TET2 (*p* < 0.05) but no significant changes in protein levels of TET3 (Figure 3E,F). In conclusion, the deletion of myostatin may promote global DNA demethylation in bovine skeletal muscle satellite cells.

### 3.3. Differentially Methylated Regions (DMRs) of the RACK1 Gene

**Figure 4:**
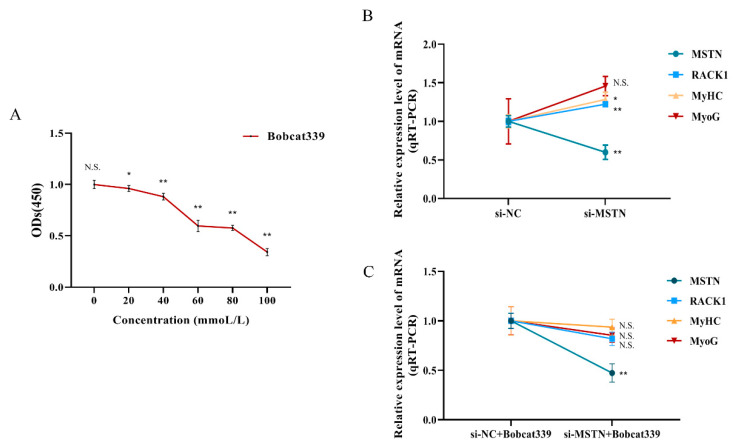
Inhibition of DNA demethylation pathways and muscle differentiation. (**A**) CCK-8 experiment to exclude Bobcat339. (**B**,**C**) The effects of Bobcat339 on RACK1, MyHC, and MyoG ((**B**) is the Bobcat339 control group, and (**C**) is the Bobcat339 experimental group). Differences between groups were statistically analyzed using Student’s *t*-test. *p* < 0.05 was considered statistically significant (* *p* < 0.05 and ** *p* < 0.01). And N.S., not significant (*p* > 0.05).

We selected the RACK1 gene (ID: ENSBTAG00000019648) as a target gene for further investigation. Deletion of myostatin resulted in increased transcript levels of RACK1 (*p* < 0.01), MyHC (*p* < 0.05), and MyoG (*p* > 0.05) (Figure 4B). We used 50 mmol/L Bobcat339 to interfere with the DNA demethylation pathway of cells (Figure 4A), which resulted in non-significant changes (*p* > 0.05) in the transcript levels of RACK1, MyHC, and MyoG, although myostatin expression was inhibited (Figure 4C). Overall, the impairment of the DNA demethylation pathway may have abolished the promoting effect of myostatin depletion on the transcript levels of RACK1, MyHC, and MyoG.

**Figure 5:**
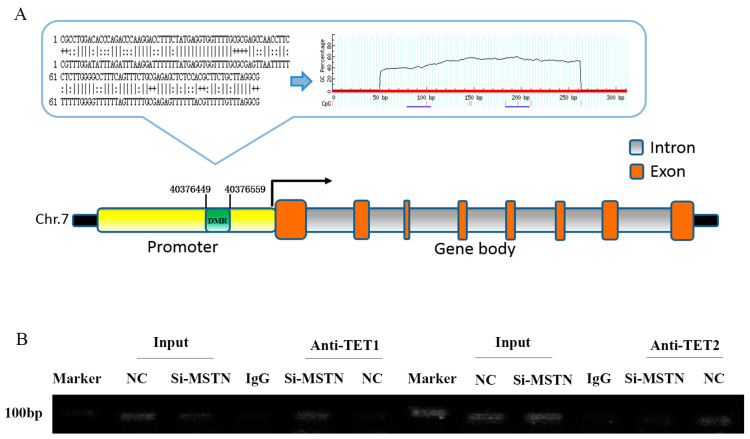
RACK1 and DNA demethylation. (**A**) Schematic representation of the DMR of the RACK1 gene. (**B**) ChIP verifies the binding of the TET1/TET2 protein to the DMR of RACK1.

RACK1 is located on chromosome 7 and has eight exons. WGBS-seq showed that its DMR contains 12 nCG sites and is located on the CpG island of the promoter (Figure 5A). We used the ChIP assay to detect the DMR-bound demethylase of RACK1 after knockdown of the myostatin gene. The results showed no significant change between the NC and si-MSTN groups by pulling down the DMR fragment with anti-TET2. However, we used anti-TET1 to pull down the DMR fragment and the si-MSTN group was brighter than the NC group, indicating a stronger accumulation of the DMR fragment (Figure 5B). In summary, the DMR of RACK1 binds more strongly to TET1 after the deletion of the muscle growth inhibitor.

### 3.4. Impact of RACK1 Overexpression on Myogenic Differentiation of Bovine Skeletal Muscle

**Figure 6:**
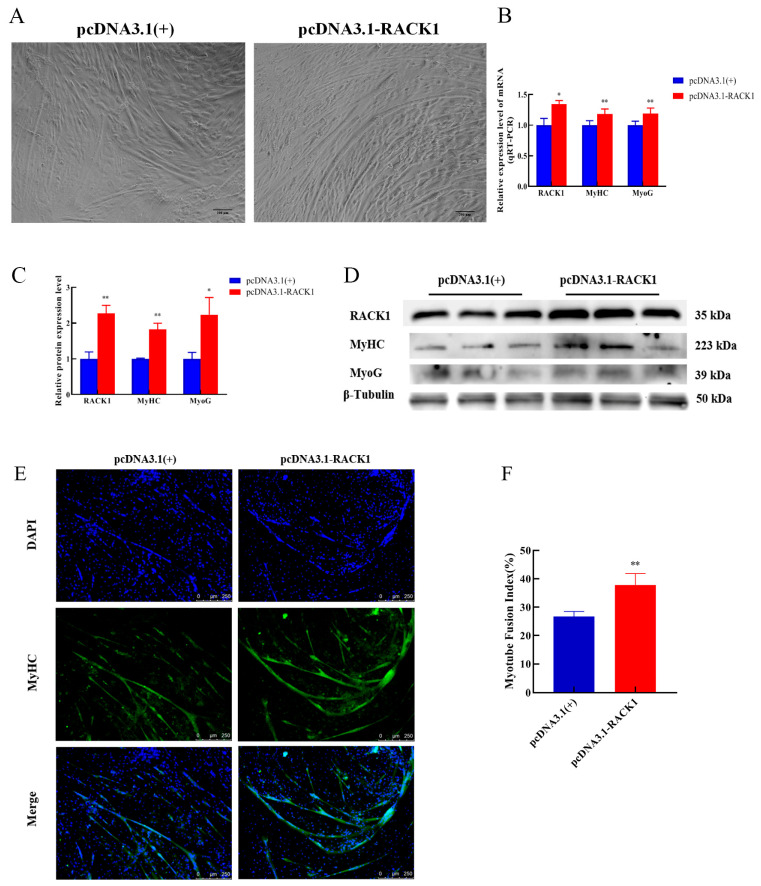
Effects of RACK1 overexpression on myogenic differentiation of bovine skeletal muscle. (**A**) Light micrographs (100×) of cells before and after overexpression of RACK1. (**B**) qRT-PCR detection of overexpression of RACK1 on muscle differentiation markers (MyHC and MyOG). (**C**,**D**) Protein levels of muscle differentiation markers (MyHC and MyOG) after overexpression of RACK1. (**E**) MyHC expression was detected by immunofluorescence staining and the nuclei were counterstained with DAPI (100×). (**F**) Myotube fusion index expressed as the number of nuclei in the myotube/total number of nuclei. Differences between groups were statistically analyzed using Student’s *t*-test. *p* < 0.05 was considered statistically significant (* *p* < 0.05 and ** *p* < 0.01). And N.S., not significant (*p* > 0.05).

Myotube morphology was observed under a light microscope (Figure 6A). Exogenously increased expression of RACK1 in cells resulted in a significant increase in the transcript levels of MyHC and MyoG (*p* < 0.01) (Figure 6B). In addition, overexpression of RACK1 increased the protein levels of MyHC (*p* < 0.01) and MyoG (*p* < 0.05) (Figure 6C,D), consistent with transcription. The immunofluorescence staining results showed that overexpression of RACK1 increased the myotube fusion index (*p* < 0.05) (Figure 6E,F). In conclusion, the increase of RACK1 has a positive influence on the myogenic differentiation of bovine skeletal muscle satellite cells.

### 3.5. Impact of RACK1 on the PI3K/AKT/mTOR Pathway

**Figure 7:**
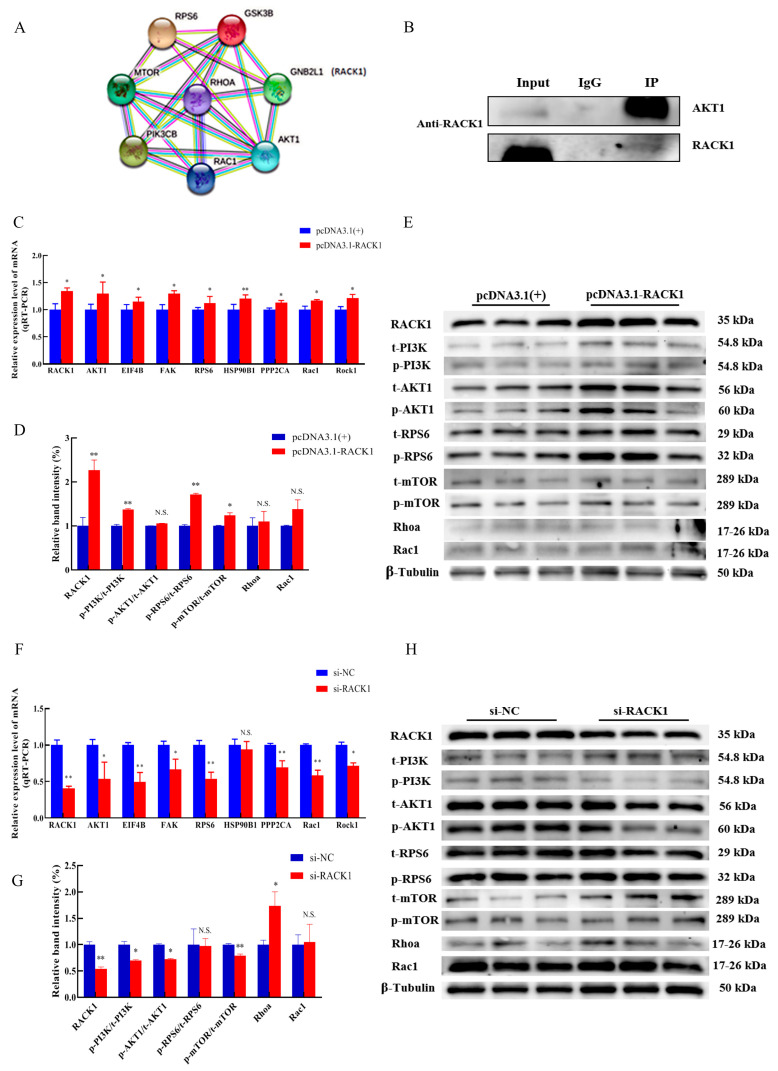
Prediction of the interaction between the RACK1 gene and the PI3K/AKT pathway. (**A**) STRING predicts the interaction between the RACK1 protein and key proteins of the PI3K/AKT pathway. (**B**) Co-IP confirms the interaction between RACK1 protein and AKT1 protein. (**C**) qRT-PCR detection of transcription levels of key genes in the PI3K/AKT/mTOR pathway after overexpression of RACK1. (**D**,**E**) Changes in expression of key proteins in the PI3K/AKT/mTOR pathway after overexpression of RACK1. (**F**) qRT-PCR detection of transcription level of key genes of PI3K/AKT/mTOR pathway after inhibition of RACK1. (**G**,**H**) Changes in the expression of key proteins of PI3K/AKT/mTOR pathway after RACK1 inhibition. Differences between groups were statistically analyzed using Student’s *t*-test. *p* < 0.05 was considered statistically significant (* *p* < 0.05 and ** *p* < 0.01). And N.S., not significant (*p* > 0.05).

The STRING software showed that the RACK1 (GNB2L1) protein interacts with key proteins AKT1, GSK3B, RPS6, and RHOA in the PI3K/AKT pathway (Figure 7A). Co-IP experiments also showed that AKT1 was significantly enriched in the immunoprecipitates of RACK1 (Figure 7B). Moreover, overexpression of RACK1 increased the transcript levels of AKT1, EIF4B, FAK, RPS6, PPP2CA, Rac1, Rock1 (*p* < 0.05), and HSP90B1 (*p* < 0.01) (Figure 7C). We observed that the total protein form (t-AKT1) and the phosphorylated form (*p*-AKT1) of AKT1 were strongly upregulated. The *p*-AKT1/t-AKT1 ratio did not change (*p* > 0.05) as the total and phosphorylated forms were similarly upregulated (Figure 7D,E). In contrast, overexpression of RACK1 resulted in an increased ratio of phosphorylated/total protein forms of PI3K (*p* < 0.01), RPS6 (*p* < 0.01), and mTOR (*p* < 0.05) (Figure 7D,E). However, RACK1 impairment had the opposite effect on PI3K/AKT/mTOR to RACK1 overexpression (Figure 7F–H). Therefore, we believe that RACK1 may have a positive regulatory effect on the PI3K/AKT/mTOR pathway.

**Figure 8:**
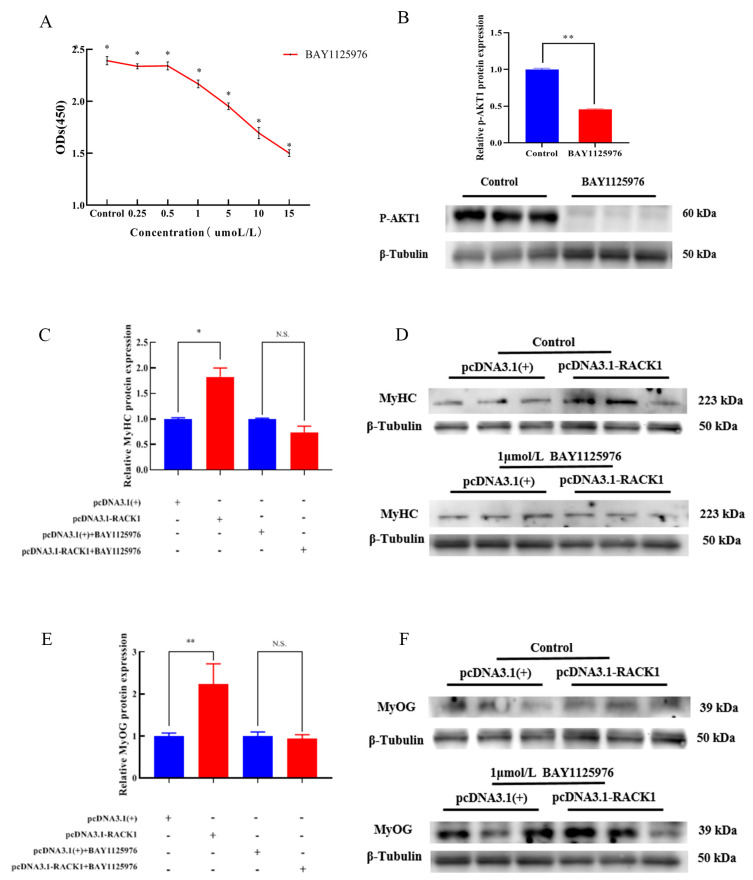
Inhibition of the PI3K/AKT/mTOR pathway affects myogenic differentiation after overexpression of RACK1. (**A**) CCK-8 assay to screen the concentration of BAY1125976. (**B**) Evidence of the inhibitory effect of BAY1125976 on AKT1 protein level. (**C**,**E**) Results of quantification of protein gray value analysis. (**D**,**F**) Representative bands of MyHC and MyOG after overexpression of RACK1. Differences between groups were statistically analyzed using Student’s *t*-test. *p* < 0.05 was considered statistically significant (* *p* < 0.05 and ** *p* < 0.01). And N.S., not significant (*p* > 0.05).

Furthermore, we confirmed the positive regulatory effect of RACK1 overexpression on the PI3K/AKT/mTOR pathway by inhibiting this pathway (Figure 8A,B). Interestingly, we found that inhibition of the PI3K/AKT/mTOR pathway did not result in significant changes in MyHC and MyoG protein levels following RACK1 overexpression (*p* > 0.05) (Figure 8C–F). In conclusion, inhibition of the PI3K/AKT/mTOR pathway can abolish the promotion of myogenic differentiation of bovine skeletal muscle satellite cells by RACK1 overexpression.

## 4. Discussion

Skeletal muscle is an economically important tissue in animals used for meat production and plays a critical role in body movement, metabolism, and maintenance of balance [20,21,22,23]. Any impairment in the transmission of a network that is interconnected and responsible for coordinating muscle growth and development can result in the loss or atrophy of muscle mass [24]. Numerous studies have shown that myostatin signaling is another important target for the treatment of muscle wasting and metabolic disorders [1,25]. In addition, specific epigenetic changes that occur during myogenesis are equally crucial for the formation and development of skeletal muscle. Among other roles, DNA demethylation contributes significantly to cellular immunity [26], cellular hypertrophy [27], and germ cell development [28]. However, the regulatory mechanism by which DNA demethylation affects bovine skeletal muscle performance remains unclear. In recent years, the WGBS-seq method has become an effective way to screen and study DNA demethylation at high throughput. Here, we use Luxi yellow cattle to study bovine skeletal muscle development. We have mapped genome-wide DNA methylation profiles to investigate the DNA demethylation landscape during bovine muscle development. In particular, we systematically studied two *MSTN*^+/−^-edited and wild-type cattle at the same growth stage to search for factors that influence bovine skeletal muscle development.

DNA methylation and demethylation of myogenesis-specific genes are critical regulatory factors for muscle satellite cell differentiation. Previous studies have shown that DNA demethylation can promote myogenesis in late myoblast hypertrophy [29] and drive myogenesis [30]. Early demethylation of myogenic genes also contributes to the premature terminal differentiation of myoblasts [31]. Active demethylation type of DNA relies mainly on the TET enzyme family. The TET enzyme generates and protects low-level methylation in the key regulatory region of the whole genome [8,9]. TET2 is obligatory for muscle regeneration in the body. TET2 activates the transcription of key differentiation and regulatory factors by activating the myogenin enhancer region to produce demethylation, which then regulates muscle cell differentiation and fusion [32]. In induced differentiated C2C12 myoblasts, the silencing of TET2 can impair myoblast differentiation [33]. Similarly, our results show that myostatin deletion resulted in the downregulation of global DNA methylation and increased transcriptional and protein levels of TET1 and TET2.

Little has been reported on the regulation of RACK1 during skeletal muscle development. We have found that deletion of myostatin leads to increased expression of RACK1. RACK1 is a protein that interacts with IGF-1R and regulates receptor signaling [34]. The anabolic effect of IGF-1 is mediated by specific binding to the IGF-1 receptor, and IGF-1 inhibits myostatin signaling during myogenic differentiation [35]. RACK1 may be involved in IGF-I signaling by forming a protein that interacts with the IGF-1R. Therefore, myostatin might regulate RACK1 through the IGF-1 signaling pathway. Our study found that the enzyme TET1 binds to the DMR of the RACK1 promoter when myostatin is inhibited, resulting in DNA demethylation and increased transcription. We believe that deletion of myostatin can also activate the activity of the TET1 promoter by silencing the SMAD2/SMAD3 pathway [36,37], and eventually affect the activity of the RACK1 promoter and promote the expression of RACK1. Furthermore, one study showed that reduced expression of RACK1 in C2C12 myoblasts significantly repressed the transcription of MyHC and MyoG [38]. Similarly, we overexpressed RACK1 in bovine skeletal muscle satellite cells and found that it promoted the expression of MyHC and MyoG.

Previous reports have shown that myostatin is a secreted protein that transmits signals to the nucleus through a series of tandem reactions. Examples include PI3K/AKT [39], Smad [40], and insulin-like growth factor-1 (IGF-1) [35]. Studies have shown that IGF-1 and insulin have an antagonistic effect on the P13K/PTEN/AKT pathway that mediates myostatin-induced p300 degradation [41]. The difference is that IGF-1 promotes protein synthesis in skeletal muscle via the PI3K/AKT/mTOR and PI3K/AKT/GSK3β pathways [42]. A novel functional crosstalk between the IGF-1 and myostatin signaling pathways has been reported, mediated by the interaction of PI3K/AKT and Smad3. Myostatin inhibits myoblast proliferation and differentiation and regulates muscle growth and metabolism through the combined action of the PI3K/AKT pathway. Similarly, RACK1 is closely linked to the PI3K/AKT pathway [43,44,45,46,47,48]. We predicted and validated the protein interaction between RACK1 and AKT1 and found that inhibition of the PI3K/AKT/mTOR pathway rescued the promotion of MyHC and MyoG through overexpression of RACK1. 

**Figure 9:**
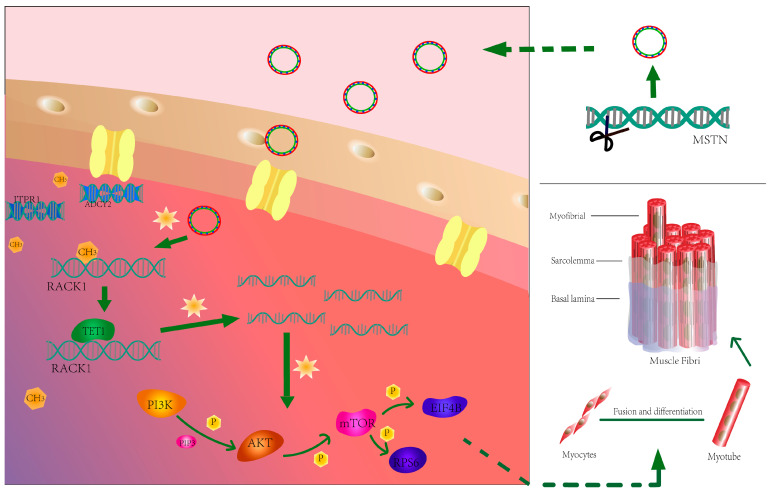
Myostatin regulates the molecular mechanism of myogenic differentiation by reducing DNA methylation. The upper right panel shows that knockdown of myostatin was achieved by constructing site-mutated vectors to transfect cells. The present work shows that deletion of myostatin triggers a decrease in global DNA methylation and promotes binding of the enzyme TET1 to the RACK1 promoter region, leading to hypomethylation and increased transcription in the RACK1 promoter region. Increased RACK1 promotes myogenic differentiation of bovine skeletal muscle satellite cells via activation of the PI3K/AKT/mTOR pathway.

Although our data suggest that the PI3K/AKT/mTOR pathway is a potential target of RACK1, it is important to highlight the diversity of signaling pathways that myostatin regulates in muscle differentiation, and future studies will focus on the fact that deletion of myostatin causes methylation changes that affect other components and signaling pathways of muscle development. In Figure 9, we summarize the major signaling pathways through which myostatin deletion-induced DNA demethylation alters RACK1 to regulate muscle development. These findings may help us understand how epigenetics influences bovine skeletal muscle development through the deletion or amplification of specific genes.

## 5. Conclusions

Previous studies have shown that the deletion of myostatin affects muscle cell proliferation and differentiation. In this study, deletion of myostatin led to global DNA demethylation, and more interestingly, knocking out myostatin also triggered demethylation of the RACK1 promoter region; and high expression of RACK1 activated the PI3K/AKT/mTOR pathway to promote myogenic differentiation. In summary, we have uncovered part of the molecular mechanism of the myostatin-mediated reduction of DNA methylation in the regulating myogenic differentiation of bovine skeletal muscle satellite cells. This provides important clues for further research into the regulatory role of myostatin and DNA methylation in skeletal muscle development.

## Figures and Tables

**Table 1:** Sequencing data by whole genome bisulfite sequencing (WGBS) for bovine *MSTN*^+/−^-edited (Z) and wild type (F).

Groups	Sample	Clean Base (Gb)	Clean Reads	GC (%)	Q30 (%)	Mapped (%)	Bisulfite Conversion Rate (%)	Total_mC (%)
Wild type	F81199	155.23	1,037,577,664	22.77	90.56	76.30	98.66	0.29
	F81208	156.91	1,049,963,834	22.93	89.09	77.00	98.84	0.37
	F81209	156.12	1,044,003,946	22.64	90.70	76.60	98.69	0.32
	F81210	155.08	1,036,046,942	22.92	88.50	76.90	98.85	0.34
	F81214	155.68	1,041,440,172	22.99	88.34	76.50	98.82	0.36
*MSTN*^+/−^-Edited	Z61004	156.31	1,046,166,382	23.17	87.40	75.68	99.20	0.36
	Z61020	155.89	1,042,125,536	23.24	90.11	74.25	98.76	0.37
	Z61023	141.55	960,468,910	30.65	87.61	59.35	99.20	0.27
	Z61117	151.37	1,016,262,024	23.29	88.51	75.24	99.20	0.34
	Z61128	155.40	1,038,158,924	22.15	87.24	76.42	99.20	0.23

Global DNA methylation analysis of *MSTN^+/−^*-edited bovine (Z) and wild-type bovine (F) muscle using WGBS with 30× genome coverage and a bisulfite conversion efficiency of 98.66–99.20%. *MSTN^+/−^*-edited bovine and wild-type bovine muscles yielded an average of 152.11 and 155.80 Giga original bases, respectively. After filtering out low-quality data, each group yielded approximately 960 million clean reads, and the Q30 range of average reads per individual ranged from 87.24% to 90.70%. The mapped reads were used for subsequent analysis as the rates ranged from 59.35% to 76.90%. All methylated genomic C sites accounted for approximately 0.33% of the total bases in each group.

## Supplementary Materials

The following supporting information can be downloaded at: https://www.mdpi.com/article/10.3390/cells12010059/s1.
