# Supplementary material for: Myostatin Mutation Enhances Bovine Myogenic Differentiation through PI3K/AKT/mTOR Signalling via Removing DNA Methylation of RACK1
Source: Cells. 2022 Dec 23;12(1):59. doi: 10.3390/cells12010059 (PMC9818849; doi:10.3390/cells12010059)
Supplement: Supplementary file 1 [file cells-12-00059-s001.zip › Supplementary Material File S1.pdf]

WGBS sequencing and library construction

Genome-wide DNA methylation profiles of gluteal muscle from Luxi yellow cattle from different treatment groups at the same stage of development were generated using the Illumina sequencing platform, and 150 bp paired-end reads were generated according to the manufacturer's standard procedures. In short, DNA was fragmented using high-pressure nitrogen gas. The TruSeq Nano DNA LT Sample Prep Kit (Illumina) processed the DNA fragments for DNA library preparation. Libraries were quantified using Picogreen and spectrofluorometer methods (Quantifluor-ST Fluorometer, Promega, E6090; Quant-iT PicoGreen dsDNA Assay Kit, P7589, Invitrogen, Carlsbad, CA). The Agilent 2100 was used for quality control of the PCR-enriched fragments to check fragment size and DNA library. distribution (Agilent 2100 Bioanalyzer, Agilent, 2100; Agilent High Sensitivity DNA Kit, Agilent, 5067-4626). Homogenise and mix multiplexed DNA libraries. Paired-end sequencing (PE) was performed on the DNA library. Each sample yielded over 1.02 billion raw reads.

Data filtering, sorting, and quality assessment

For this sample, the exit data was obtained by base calling. The exit data were stored in paired-end FASTQ format (FastQC: http://www.bioinformatics.babraham.ac.uk/projects/fastqc). The raw data from base quality sequencing was assessed using the quality value. The quality value, referred to as the Q-value (Phred quality score), is the rounded mapping result of the base reading error rate p. The correspondence between the Q-value and the p-value is calculated using the standard Sanger variant calculation method. The corresponding formula between the Q-value and the p-value is: $Q_{phred} = -10\log_{10}P$

The quality coding of the bases in the FASTQ files obtained from different sequencing platforms follows different schemes. The corresponding relationship between the Q-value and the quality information of the bases is the Q-value plus an offset value. The results are converted into the corresponding characters according to the code table ASCII. The reference information is as follows (http://en.wikipedia.org/wiki/FASTQ _format).

Statistics on the logout data of each sample, including sample name, reads, total number of bases, Q30, percentage, and Q20 (%) and Q30 (%) values. Raw data obtained from sequencing must assess sequencing quality based on the quality value. Reads with an average quality value below Q20 are removed and the qualified data are used for subsequent analysis.

Genome Annotation Database Collation and Mapping Alignment

Organise statistically the number of each gene, the features on the chromosome (number, start site, stop site, length, transcription direction), gene name, gene classification and annotation. The alignment of mapping is that Bismark aligns reads to the genome using Bowtie2, identifies base conversion events, and finally classifies and counts them.

Methylation event detection and statistics

Methylation sites of alignment results were detected with Bismark. Remove repetitive reads (usually caused by PCR), remove the repetitive part in the double-ended sequence andthen measure the DNA methylation site corresponding to the base type on the C-site alignment on the reference genome. If the base on the alignment is C, methylation has occurred; if the base on the alignment is T, no methylation has occurred. Using the Bismark statistical results, obtain the methylation context information (CpG, CHH, CHG) in all reads aligned to the genome and complete the methylation statistics from CG. Calculate the number of mCs and non-mCs and the degree of methylation of the DNA methylation sites that detected. The frequency of cytosine sites under each classification of methylated cytosine context in the relative position of reads was counted and the frequency distribution map was drawn from the 5' end to the 3' end of reads, and the biassed statistics of reads sequencing sites were performed. The chromosome-level methylation density distribution map was constructed based on the proportion of methylated sites in the C sites covered by reads on each chromosome and the average methylation level of the methylated sites. Finally, the average ratio of methylation sites in each C-content in each functional gene region was calculated (the promoter region is the region 2kbp upstream of the TSS site, which was determined by EXON and INTRON based on the structural annotation information of the genome).

Genome-wide mapping of methylation circles and identification

The motifs of methylation distribution in the genome and on CG was mapped using Circos software. Logo plots were drawn for different C contents and different methylation ratios. The level of CpG methylation was greater than 75%, and the level of non-CpG methylation was greater than 25% as hypermethylation level.
