# Supplementary material for: Myostatin Mutation Enhances Bovine Myogenic Differentiation through PI3K/AKT/mTOR Signalling via Removing DNA Methylation of RACK1
Source: Cells. 2022 Dec 23;12(1):59. doi: 10.3390/cells12010059 (PMC9818849; doi:10.3390/cells12010059)
Supplement: Supplementary file 1 [file cells-12-00059-s001.zip › Supplementary Material File S11.pdf]

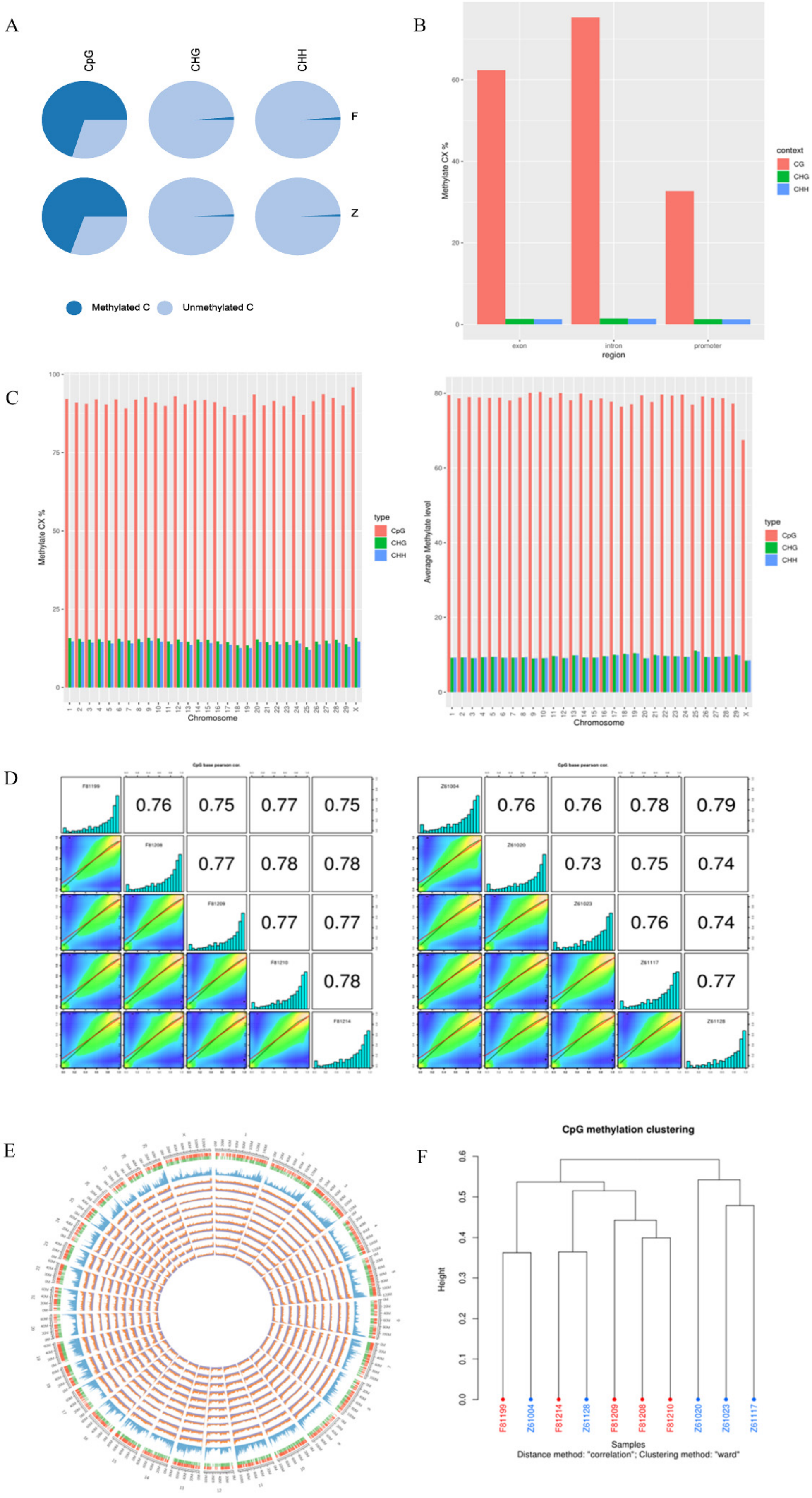
A
CpG
CHG
CHH
F
Z
Methylated C
Unmethylated C
B
Methylate CX %
context
CG
CHG
CHH
exon
intron
promoter
region
C
Methylate CX %
type
CpG
CHG
CHH
Chromosome
Average Methylate level
D
CpG base pearson cor.
0.76 0.75 0.77 0.75
0.77 0.78 0.78
0.77 0.77
0.78
0.76 0.76 0.78 0.79
0.73 0.75 0.74
0.76 0.74
0.77
E
F
CpG methylation clustering
Height
F81199
Z61004
F81214
Z61128
F81209
F81208
F81210
Z61020
Z61023
Z61117
Samples
Distance method: "correlation"; Clustering method: "ward"

Supplementary Materials S11. Genome-wide DNA methylation profiling. A, Statistics of genome-wide DNA methylation cytosine (CG, CHH and CHG) levels. Distribution of methylation levels of B, C loci in each gene functional region (exons, introns, and promoters). C, The mean methylation level (right) and proportion of methylated C sites (left) for chromosomally distributed methylated C sites. D, Correlation of sample methylation levels in wild-type (F) (left) and MSTN+/- edits (Z) (right). E, Methylation circle map. F, Cluster map of sample methylation.

The CG, CHH, and CHG (where H stands for A, C, or T) have different levels of methylation. We found genome-wide methylated cytosines (mC) of 69.74±1.06% for CG in $MSTN^{+/-}$ edited samples, 0.94±0.21% for CHG, 0.94±0.21% for CHH, and 70.34±1.26% for CG in wild-type samples, CHG was 1.12 ± 0.26%, CHH was 1.04 ± 0.24%, and the proportions of these contexts were similar in each group (Supplementary Material S11, A). We analysed the methylation status of three different regions, including promoters, exons and introns. The CG context plays an important role and is most pronounced in introns. The CHG and CHH contexts remain ultra-hypomethylated and stable in all functional elements (Supplementary Material S11, B). Furthermore, the methylation of the CG context is evenly distributed across the genome. The samples were highly correlated (Supplementary Materials S11, C, D, E and F).
