# Supplementary material for: Myostatin Mutation Enhances Bovine Myogenic Differentiation through PI3K/AKT/mTOR Signalling via Removing DNA Methylation of RACK1
Source: Cells. 2022 Dec 23;12(1):59. doi: 10.3390/cells12010059 (PMC9818849; doi:10.3390/cells12010059)
Supplement: Supplementary file 1 [file cells-12-00059-s001.zip › Supplementary Material File S2.pdf]

Differentially methylated regions (DMRs) and their gene enrichment analysis

Differentially methylated regions (DMRs) were determined by the modest dispersion shrinkage method of DSS software (Park and Wu, 2016), which combines spatial correlation, site read depth, and variance among biological replicates and accurately detect DMRs to improve the statistical test.

Based on the distribution of DMRs in the genome, a functional analysis was performed on the genes whose gene body region (from TSS to TES) and gene promoter region (2kbp upstream of the transcription start site) overlapped with DMR. For the GO enrichment analysis (DAVID 6.8: https://david.ncifcrf.gov/) of the differentially expressed genes, GO was classified according to molecular function MF, biological process BP and cellular component CC, and the top 10 GOs with the smallest p-value, i.e. the most significant enrichment, were selected in each GO classification. The term entry is displayed. By counting the number of differentiated genes covered at the different levels of each KEGG (https://www.kegg.jp/) pathway, the metabolic pathways and signalling pathways in which the differentiated genes are mainly involved are identified.

In this study, the swDMR software (http://122.228.158.106/swDMR/) was used to identify DMRs across the genome using a sliding window approach. The window size and step size were set to 1000 and 100 bp, respectively. The resulting DMR was determined using Fisher's test. The workflow essentially involves the following:

(1) Set the sliding window size (1000 bp) and step size (100 bp), and select the region where the difference or fold change in methylation level is greater than the cutoff value and the number of cytosines contained in that region is greater than the cutoff value.

(2) The software integrates several useful statistical methods to test for significant differences, and regions with significant differences are considered potential DMRs.

(3) Hypothesis test the methylation information for the next window with a specific step size.

(4) Repeat the above steps to obtain potential DMRs across the genome.

(5) Adjust all p-values using the FDR value.

(6) Combine the overlapping potential DMRs into one region and run hypothesis

tests again. The combined DMR is considered final.
