# Supplementary material for: Myostatin Mutation Enhances Bovine Myogenic Differentiation through PI3K/AKT/mTOR Signalling via Removing DNA Methylation of RACK1
Source: Cells. 2022 Dec 23;12(1):59. doi: 10.3390/cells12010059 (PMC9818849; doi:10.3390/cells12010059)
Supplement: Supplementary file 1 [file cells-12-00059-s001.zip › Supplementary Material File S3.pdf]

Pyrosequencing experiment

Methylation was performed using the QiagenEpiTect Bisulfite Kit (Qiagen, GER).

(1) Methylation modification

Prepare a total system of 140 μL, including 85 μL of sulfite mixture, 15 μL of DNA protection solution, DNA solution (1–500ng, up to 40 μL), and RNase-free water supplement. Add 800 μL of enzyme-free water to the mixture of sulfite and DNA, mix by pipetting, and place at room temperature after mixing. The PCR reaction conditions were set as denaturation at 95°C for 5 min, renaturation at 60°C for 25 min, denaturation at 95°C for 5 min, renaturation at 60°C for 1h25min, denaturation at 95°C for 5min, renaturation at 60°C for 2h55min, and hold at 20°C for variable.

(2) Purification of sulfite-modified DNA

Centrifuge the reaction solution and transfer it to a new EP tube, add 560 μL of freshly prepared Buffer BL (containing 10 μg/mL carrier RNA), and mix well. The solution was transferred to a column containing EpiTect spin columns, centrifuged for 1 min, and the filtrate was discarded. Add 500 μL of Buffer BW to the column, centrifuge for 1 min, and discard the filtrate. Add 500 μL of Buffer BD to the column, incubate at room temperature for 15 min, centrifuge for 1 min, and discard the filtrate. Add 500 μL of Buffer BW to the column, centrifuge for 1 min, and discard the filtrate. repeat. The column was taken out and placed in a new EP tube, and centrifuged for 1 min. Open the lid and put it in a new EP tube, and react at 56 °C for 5 min. Take out the column and put it in a new EP tube, add 20 μL of Buffer EB dropwise, centrifuge for 1 min, and save the eluted product.

(3) Primer design

The primer design software used was PyroMark Assay Design 2.0. See Supplementary table 1 for primer sequence information.

(4) PCR amplification

PCR amplification system (50μL): 34.8μL H_2O, 10μL 5×bufferGC (KAPA), 1μL dNTP (10mM/each), 1μL Primer (up 50pM/ul), 1μL Primer (down 50pM/ul), 2μL Template, 0.2μL Taq (5U/μL). PCR program (40Cycles): 95℃ 3min, 94℃ 30sec, 56℃ 30sec, 72℃ 1min, 72℃7min, 4℃ ∞.

(5) Pyrosequencing Assay - Q96

Configure the reaction system (2 μL reaction binding beads, 38 μL binding buffer and 40 μL PCR product) in a 96-well PCR reaction plate, and mix by pipetting for 10 min. The vacuum pump sucked the binding beads and the mixed solution, and then immersed them in ethanol (70%), NaOH (0.2M) and washing buffer in an orderly manner for 5s each. Put the binding beads and PCR products on the probe into 40 μL of annealing buffer, denature at 85°C for 2 min, and cool to room temperature. Calculate the configuration system and add substrate mix, enzyme mix and dNTPs in sequence in the reagent compartment. Put the reagent chamber and 96-well reaction plate into the Pyrosequencing detector to start the reaction.

(6) Pyrosequencing detection-Q48

Sets the running program of the PyroMark Q48 software. Import the program into a USB flash drive and plug it into the PyroMark Q48's USB port. Click "Sequence" displayed on the touch screen of the instrument to load the running program in the U disk. Put in the cotton sliver that absorbs waste liquid, and ensure that the end-to-end connection is in the positive left direction. Push open the cover of the instrument, open the cover of each card holder and select the card holder that needs prime, the selected card holder will display the water drop icon. Select whether this run is the last run. Reagents are added to each cartridge sequentially according to the volume of each reagent displayed on the touch screen. When adding the reagent to the cartridge, use the sample pipetting gun to add slowly to the wall to avoid air bubbles. After adding the reagents, close the lid of the cartridge and lock it. Click "Start" to test whether the function of the card holder is normal. A green arrow will appear behind the card holder that has passed the test. Place the loading disc into the machine, taking care to align the well-located holes on the disc. Tighten the set screw that secures the sample disc. Shake the magnetic beads, and then add the magnetic beads to the sample disk using the continuous sample gun delivered by the machine. Each well is 3 μL, and 3 wells can be added continuously at one time. Add 10μL of biotin-labeled PCR product to the well of the loading disc. If the PCR product is not enough to 10μL, make up to 10μL with pure water to ensure that the PCR product liquid completely covers the magnetic beads. The Q48 program was run, and the final pyrosequencing results were analyzed.

Supplementary table 1

Supplementary table 1: Primers of pyrosequencing

Primer Name	Sequence (5'-3')	5' Modification
ADCY2-F	GGAAGTTGAGGATATTGTTTTATGAGTAGA	Biotin
ADCY2-R	ATACACCTACCCTCAAACACTAATAATCC	
ADCY2-S	CAAACACTAATAATCCCC	
ITPR1-1F	AGTAGTTGTTAGGAATATTAGTAGTGT	Biotin
ITPR1-1R	TCTAATCATATTATCCCTTCCTTCTT	
ITPR1-1S	AGGAATATTAGTAGTGTTTGA	
ITPR1-2F	AGGAAGGGATAATATGATTAGAAGAA	Biotin
ITPR1-2R	AACAAATATTACCTACAACCTCTTC	
ITPR1-2S	TTTTTAGTTTGTGTTGGAAT	
BDKRB2-1F	TTGGGGAAGAGGGGTTGAAA	Biotin
BDKRB2-1R	CCCAAAACTCCTCCTAACTTCT	
BDKRB2-1S	GGGAAGAGGGGTTGAAAA	
BDKRB2-2F	AGGGTGGTTGTAGTTGTTTAGG	Biotin
BDKRB2-2R	ACCCAAACCTCTCAAAAACAACCTAT	
BDKRB2-2S	ACAACCTATCTACACAAT	
RACK1-1F	GTTTTTGGGTTTGGGTGTT	Biotin
RACK1-1R	CCACACCATATAAAATAAATACTATTATTC	
RACK1-1S	GGGTTTGGGTGTTTAG	
RACK1-2F	TGGGTAAGTTTGGATATTTAGATTTAAGGA	Biotin
RACK1-2R	CTACTAAACAATAAACCCCACTCC	
RACK1-2S	GATTTTTTTATGAGGTGGT	
