# Supplementary material for: Myostatin Mutation Enhances Bovine Myogenic Differentiation through PI3K/AKT/mTOR Signalling via Removing DNA Methylation of RACK1
Source: Cells. 2022 Dec 23;12(1):59. doi: 10.3390/cells12010059 (PMC9818849; doi:10.3390/cells12010059)
Supplement: Supplementary file 1 [file cells-12-00059-s001.zip › Supplementary Material File S4.pdf]

Bovine skeletal muscle satellite cells resuscitation and induction of differentiation culture

The bovine skeletal muscle satellite cells were provided by the Tianjin Agricultural Animal Breeding Health and Healthy Cultivation Centre. Bovine skeletal muscle satellite cells were isolated from foetal bovine limb muscle and stored in the laboratory. They were taken out and placed in a 37 ℃ constant temperature when they were taken out again. The tube for cryopreservation of cells must be shaken rapidly until the frozen liquid melts. When the cryopreservation liquid melts, an equal volume of proliferation medium has been added to neutralise it. Centrifuge at 1000 rpm for 10 minutes. The medium was then drained and the cell precipitates were suspended by the proliferation medium. The cells were inoculated into Petri dishes at a density of 30% and homogenised in ∞- type and cultured in 37℃ 5% CO_2 incubator. When the cell density reached about 80%, the medium was settled and 1mL of 0.25% trypsin (Solarbio, Beijing, China) was added. The cells were digested in a CO_2 incubator at 37℃ for about 2 minutes. An equal volume of medium was then added to stop the digestion process. The centrifuge was set at 1000 rpm for 10 minutes. Proliferation medium: 10% FBS (Gibco)+ 90% DMEM (Gibco, USA). Differentiation medium: 2% HS (Gibco, USA) + 98% DMEM (Gibco, USA).

cell transfection

The cell transfection steps were performed according to the Lipofectamine 3000 (Invitrogen, Carlsbad, CA) instructions. Using a 6-well plate as an example, transfection was performed when cell density reached 65% and 3 biological replicates were set for each group. Dilute Lipofectamine 3000 and siRNA/si-NC with Opti-MEM medium in the following ratio: (1) 100 μL opti-MEM + 6 μL lipofectamine3000. (2) 100 μL opti-MEM + 10 μL siRNA/si-NC. (1) and (2) were incubated at room temperature for 5 minutes, then added and mixed and incubated at room temperature for 15 minutes. Aspirate and discard 200 μL of the culture medium from each well, add 200 μL of the siRNA complex prepared above and mix gently; culture the cells at 37°C in a 5% CO_2 incubator for 72 hours after induction of differentiation as evidence of the third day of cell differentiation point.

Immunofluorescence staining

MyHC immunofluorescence staining was performed on the cells on the third day of induced differentiation, taking a 48-well plate as an example, and three biological replicates were set for each group. Aspirate the liquid and wash the cells twice with

PBS. 100 μL of 4% paraformaldehyde (Solarbio, Beijing, China) was added to each well, fixed for 30 min, aspirated and discarded, and washed 3 times with PBS. Pipette 100 μL of Triton X-100 (Solarbio, Beijing, China) into each well, the cells are permeabilized for 20 min, aspirated and discarded, and washed 3 times with PBS. Add 100 μL of 5% BSA (Solarbio, Beijing, China) to block for 30 min, and remove the blocking solution. Add 100 μL of primary antibody (anti-MyHC, DSHB, USA, diluted 1:500 with 1% BSA) and remove the primary antibody after overnight at 4°C in a humidified chamber. Wash 3 times with PBS, add the corresponding goat anti-mouse IgG-FTIC (1:500 with 1% BSA, Solarbio, Beijing, China), incubate at 37°C for 1 h in the dark, aspirate and discard, and wash 3 times with PBS. Pipette 100 μL of DAPI (Solarbio, Beijing, China) into each well, stain in the dark for 5 min, aspirate and discard, add 100 μL of PBS, and image with a fluorescence microscope. At least 3 different fields of view are taken for each biological replicate.
