# Supplementary material for: Myostatin Mutation Enhances Bovine Myogenic Differentiation through PI3K/AKT/mTOR Signalling via Removing DNA Methylation of RACK1
Source: Cells. 2022 Dec 23;12(1):59. doi: 10.3390/cells12010059 (PMC9818849; doi:10.3390/cells12010059)
Supplement: Supplementary file 1 [file cells-12-00059-s001.zip › Supplementary Material File S5.pdf]

Extraction of total cellular RNA

Total cell RNA was extracted using the EASYspin Plus tissue/cell RNA rapid extraction kit (Aidlab Biotechnologies Co., Ltd, Beijing, China). The entire process was performed in a sterile environment using a 24-well plate as an example. Remove the Petri dish from the incubator, observe and photograph under the microscope to record the condition of the cells. Discard the liquid, wash the cells (pre- cooled with enzyme-free PBS), add 350 μL RLT Plus to each well to lyse the cells and transfer them to an enzyme-free EP tube. Transfer the liquid to a DNA purification column, centrifuge at 13,000 rpm for 1 minute, and discard the purification column. Immediately add an equal volume of 70% ethanol and mix well. 700 μL of the mixture was added to the RA column, centrifuged at 13,000 rpm for 30 s and the filtrate removed. Add 700 μL of the deproteinised solution RW1 to the adsorption column, leave for 1 minute, centrifuge at 12000 rpm for 1 minute, and discard the waste liquid. Add 500 μL of the rinsing solution RW to the adsorption column, centrifuge at 12,000 rpm for 1 minute, and discard the waste liquid. Repeat this procedure once. Replace with a new collection tube and centrifuge at 13,000 rpm for 3 minutes. Add the adsorption column RA to a new enzyme-free tube, add 35 μL of pre-warmed enzyme-free water to the adsorption membrane, leave for 3 minutes and centrifuge at 12,000 rpm for 2 minutes. Repeat this procedure to increase the collection concentration.

Synthesis of cDNA First Strand

The first strand of cDNA was synthesized using HiFiScript cDNA Synthesis Kit (CWBIO, Jiangsu, China). The specific operation steps are as follows:

(1) The first-strand cDNA synthesis reaction system was prepared as shown in Table 1, and mixed by pipetting.

Table1 cDNA first chain synthesis reaction system

Reagent	Volume (μL)
dNTP Mix (2.5μM Each)	4.0
Primer Mix	2.0
DTT (0.1M)	2.0
5×RT Buffer	4.0
HiFiScript，200U/μL	1.0

RNA Template	50pg~5μg
RNase Free dH_2O	Add to 20μL

(2) PCR reaction program settings: 42°C, 15minutes; 85°C, 5minutes; short-term storage at 4°C, and frozen at -20°C.

Real-time quantitative PCR (qRT-PCR)

All primers for qRT-PCR experiments are detailed in Table 2. The system configuration (Table 2) and procedures (Table 3) are as follows.

Table 2 qRT-PCR reaction system

Reagent	Volume (μL)
2×All-in-One^{TM}qPCR Mix (GeneCopoeia)	10.0
Forward primer	1.0
Reverse primer	1.0
cDNA template	2.0
RNase Free dH_2O	Add to 20μL

Table 3 qRT-PCR reaction program

Procedure	Temperature	Time	Cycles
Preincubation	95℃	600s	1
3 Step Amplification	95℃	10s	35
	60℃	15s	
	72℃	15s	
Melting	95℃	10s	1
	65℃	60s	
	97℃	1s	
Cooling	37℃	30s	1

Supplementary table 2

Supplementary table 2 Primers of qRT-PCR

Genes		Primer sequence (5’-3’)	Product length （bp）
MSTN	F	TGTGGAAAAAGAGGGGCTGT	112
	R	AGCTGTTTCCAGGCGAAGTT	
MyHC	F	TGCTCATCTCACCAAGTTCC	105
	R	CACTCTTCACTCTCATGGACC	
MyoG	F	CAAATCCACTCCCTGAAA	140
	R	GCATAGGAAGAGATGAACA	
TUBB	F	CAACAGCACAGCCATCCAGGA	131
	R	TCTCAGCCTCGGTGAACTCCAT	
FAK	F	TGGCTGCTGCTTACCTTGACC	86
	R	AGAACGCTCCACACCAGTCC	
Rock1	F	ACCAGGAAGGTGTACGCTATGA	96
	R	GCTGAACAACCCAAGGACTGT	
Rac1	F	TCCCAACACACCCATCATCCT	93
	R	GGCGTCAGCTTCTTCTCCTTC	
RPS6	F	CGAATCAGTGGCGGGAACGATA	84
	R	ACTCAGTAGCAGGCGAACTCTG	
EIF4B	F	TCGTGATGGGTATCGGGACAGT	98
	R	AGTCTCTGCCACCTCGGTCAT	
HSP90B1	F	GAGTCTCCGTGTGCTCTGGTAG	94
	R	TGTCCTTGCCTGTCTGGTATGC	
PPP2CA	F	GGGTCCAATGTGTGACTTGC	92
	R	CCTGCCCAAAGGTGTAACCA	
AKT1	F	ATTACCTGCACTCGGAAAAGGAA	
	R	CGGTGATCTTGATGTGCCCG	
RACK1-promoter	F	CCTGGACACCCAGACCCAA	86
	R	TCTCGCAGAAACTGAAAGG	

RACK1	F	GGATGGGCAGGCTATGTTGT	
	R	GAGCCAGTAGCGGTTAGGAC	
TET1	F	GTCTGGTTCGACAGCGTACA	171
	R	CATTGAGCGTGCATCTTCGG	
TET2	F	AACCAAGCAAATGCGTTCCC	193
	R	TGGATCCAGGCTCGACCTTA	
TET3	F	GGACTCTGCCTTCTGGTGAC	187
	R	GAGGAGAGTTGTGTGAGGGC	
DNMT1	F	AGGATGACCCATCTCCCGAA	375
	R	CCCCAGCCCGATCATAACTC	
DNMT2	F	CAGCGATCTCTCTGTGCGAA	388
	R	TCCAAGTAGACGGTAACGCTG	
DNMT3A	F	AGAAAGCCAAGGTGATCGCA	210
	R	CCTCGTACTCCGGTTCATCG	
DNMT3B	F	GACAAGCACGCCAACAGAAG	188
	R	CTGGAGACCTCCCTCTTGGA	
