# Supplementary material for: Myostatin Mutation Enhances Bovine Myogenic Differentiation through PI3K/AKT/mTOR Signalling via Removing DNA Methylation of RACK1
Source: Cells. 2022 Dec 23;12(1):59. doi: 10.3390/cells12010059 (PMC9818849; doi:10.3390/cells12010059)
Supplement: Supplementary file 1 [file cells-12-00059-s001.zip › Supplementary Material File S6.pdf]

Western blot analysis

Cells were lysed with PMSF (Sohrab, Beijing, China) RIPA buffer (Takara, Dalian, China) and total protein was collected. The total protein concentration of the extract was determined by BCA (CWBIO, Beijing, China). Then an equal amount of total protein was separated with 12.5% SDS-PAGE (Epizyme Biotech, Shanghai, China) and transferred to a polyvinylidene fluoride membrane (Millipore, Burlington, MA). The membrane was blocked with 5% skimmed milk powder for 1 hour and incubated overnight at 4°C with the primary antibody. It was then incubated with the secondary antibody for 2 hours and detected with the chemiluminescent ECL substrate (Sorabi, Beijing, China). For quantitative analysis using Image Lab software, the fold change of protein was normalised to GAPDH or β-tubulin (Abmart, Shanghai, China) used for western blot analysis are as follows: Anti-AKT1, Anti-Rock1, Anti-TET2 (Abcam, UK); TET2 antibody (HCLC, Thermo Fisher, GER); Anti-MyoG, Anti-MyHC (DSHB, USA); Anti-TET1, Anti-TET3, Anti-RACK1, Anti-p-AKT1, Anti-p-RPS6, Anti-PI3K, Anti-p-PI3K, Anti-mTOR, Anti-p-mTOR, Anti-GNB2/RACK1 (Abmart, Shanghai, China); Anti-RPS6 (Sangon Biotechnology Co., Ltd. Shanghai, China); Anti-Racl, Anti-RhoA (New East, USA).

CCK-8 analysis

Cells were grown in 96-well cell culture plates. Each well was loaded with 100 μL of cell suspension and cultured at 37°C in a 5% CO2 incubator for 24 hours. Add 10 μL of different concentrations of test compounds (Bobcat339, Selleckchem; BAY1125976, GLPBIO, USA) to each well. Incubate for 24 hours at 37°C in a 5% CO2 incubator. Add 10 μL CCK-8 (HANBIO, Shanghai, China) solution to each well and continue incubation for 4 h. Measure the absorbance of the sample using a microplate reader (set the wavelength to 450 nm).
