# Supplementary material for: Myostatin Mutation Enhances Bovine Myogenic Differentiation through PI3K/AKT/mTOR Signalling via Removing DNA Methylation of RACK1
Source: Cells. 2022 Dec 23;12(1):59. doi: 10.3390/cells12010059 (PMC9818849; doi:10.3390/cells12010059)
Supplement: Supplementary file 1 [file cells-12-00059-s001.zip › Supplementary Material File S7.pdf]

Ch-IP experiment

Ch-IP experiments were performed according to the instructions of the the Chromatin Immunoprecipitation (ChIP) Assay Kit (Beyotime Biotechnology, Shanghai, China).

(1) Optimise of sample collection and sonication conditions

Incubate and mix SDS Lysis Buffer appropriately. Collect cell samples on the third day of differentiation, add 270 μL 37% formaldehyde to a 10 cm cell culture dish, shake gently to distribute evenly, and then incubate at 37°C for 10 minutes. Add 1.1 mL glycine solution (10X), shake gently to distribute evenly, and leave at room temperature for 5 minutes. Place the Petri dish on an ice bath and discard the liquid. Add 8 mL of pre-chilled PBS (containing 1 mM PMSF, ready-to-use) and wash the cells twice. Add 1 mL of pre-chilled PBS with 1 mM PMSF and scrape the cells with a cell scraper into a new EP tube. Count the cells and divide them into aliquots (about 1 million per tube). Centrifuge at 800-1000 × g for 3 minutes at 4°C. Discard the supernatant. Add 200 μL SDS Lysis Buffer (containing 1 mM PMSF) to resuspend the cells. Place the cells in an ice bath for 10 minutes to complete lysis. Add 200 μL of the sample to an ice bath for sonication. Programme settings (2 mm ultrasound head): Power is 80-90 W, total time 3 minutes, ultrasound on for 4 s, and ultrasound off for 3 seconds. Fragment the genomic DNA so that the fragment size is in the range 200-1000bp. 8 μL 5M NaCL was added to the samples after sonication. Incubate at 65°C for 4h. 4 μL of the purified DNA fluid was taken and subjected to agarose gel electrophoresis to determine the efficiency of sonication fragmentation of genomic DNA.

(2) Chromatin immunoprecipitation

After sonication, samples were centrifuged at 12000-14000 × g for 5 min at 4°C. Transfer the supernatant to a 2mL EP tube. Prepare 1.8 ml of ChIP dilution buffer (1mM PMSF) per sample to dilute the sample to a final volume of 2 mL. Take 20 μL of the above liquid as input (positive control) and save it. Add 70 μL of protein A+G agarose/salmon sperm DNA to the remaining samples and mix at 4°C for 30 minutes at 4°C. Centrifuge at 1000 × g for 1 min at 4°C, and transfer the supernatant to a new 2 mL EP tube. Add 2-4 μL of the primary antibody. Swirl overnight at 4°C (IgG as negative control). Add 60 μL of the protein A+G agarose/salmon sperm DNA

precipitation complex, shake and mix at 4°C for 1 h. Centrifuge at 1000 × g for 1 min at 4°C. Discard the liquid (do not touch the pellet). The precipitated complexes were then washed with the following solution of 1 mL, washed with shaking at 4 °C for 5 min, and then centrifuged at 1000 × g for 2 min at 4 °C. Discard the liquid (do not touch the pellet).

a. Low Salt Immune Complex Wash Buffer 1 wash.

b. High Salt Immune Complex Wash Buffer 1 wash.

c. LiCl Immune Complex Wash Buffer 1 wash.

d. TE Buffer 2 wash.

(3) PCR amplification of the target gene sequence

Prepare the elution buffer (1% SDS, 0.1M NaHCO3, Beyotime Biotechnology, Shanghai, China), ready for use. After washing, add 250 μL of elution buffer. Vortex to mix, set aside at room temperature, and continue washing for 5 minutes with shaking. Centrifuge at 1000 × g for 1 minute, and transfer the supernatant to a new EP tube. Repeat the washing procedure by adding 250 μL elution buffer to the pellet. Centrifuge at 1000 × g for 1 minute, and transfer the liquid into an EP tube. Add another 20 μL 5M NaCl (add 1 μL 5M NaCl to the 20 μL Input sample) and mix by pipetting. Heat at 65°C for 4 hours to remove binding of protein and genomic DNA. Purify the samples: Add 20 μL 1M Tris pH 6.5, 10 μL 0.5M EDTA and 1 μL 20 mg/mL proteinase K to the sample. Mix by pipetting and heat the sample at 45°C for 1 h. Sample purification was performed using PCR/DNA purification kit (D0033, Beyotime Biotechnology, Shanghai, China). Input samples do not have to be purified. The purified samples were subjected to PCR experiments. The system (Table S7-1) and procedure (Table S7-2) were as follows:

Table S7-1 PCR reaction system

Reagent	Volume (μL)
2×Es Taq MasterMix (Dye)	10.0
Forward primer	1.0
Reverse primer	1.0
DNA template	2.0
RNase Free dH_2O	Add to 20μL

Table S7-2 PCR reaction program

Procedure	Temperature	Time	Cycles
predenaturation	94℃	5min	
denaturation	94℃	30s	35
Annealing	54℃	30s	
Extension	72℃	1min30s	
Extension	72℃	10min	

CO-IP experiment

Use Pierce Crosslink Magnetic IP/Co-IP Kit (Thermo-scientific, GER) to conduct CO-IP experiments. The specific operation steps are as follows:

(1) Preparation of cell lysate

Culture untreated bovine skeletal muscle satellite cells to the GM phase, discard the liquid, wash the cells with enzyme-free PBS (pre-chilled), and repeat once. Add the lysis buffer mixture (containing protease inhibitors) to the Piece lysis/rinse buffer at a ratio of 10:1, pipette 0.5 mL into the culture well, and incubate on ice for 5 min. Transfer the lysate to a new 1.5 mL enzyme-free EP tube and centrifuge at 13,000 rpm for 10 min at 4°C. Transfer the supernatant to a new enzyme-free EP tube.

(2) Bind the antibody to protein A/G magnetic beads

Prepare a new EP tube, add 0.1 mL of Pierce Lysis/Wash Buffer and 20× Coupling Buffer, make up to 2 mL with ultrapure water to make 1× Coupling Buffer. Vigorously shake the Pierce Protein A/G Magnetic Bead Bottle to fully suspend the magnetic beads. 25 μL of magnetic beads were added to a 1.5 mL EP tube and placed on a magnetic stand for 1 min, and the supernatant was removed. Take 0.5 mL of 1× coupling buffer and add it to the EP tube, slowly invert and mix for 1 min, place it on a magnetic stand, and remove the supernatant. Repeat 1 time. To calculate a final antibody concentration of 5 μg/100 μL, dilute the MSTN primary antibody with 20:1 Pierce Lysis/Wash Buffer and 20× Conjugation Buffer. Add 100 μL of antibody diluent to the EP tube and shake slowly for 15 min to suspend the magnetic beads. Place on a magnetic stand and discard the liquid. Take 100 μL of 1× coupling buffer into the EP tube and mix well. Place on

a magnetic stand and remove the supernatant. Aspirate 300 μL of 1× coupling buffer into the EP tube and mix well. The magnetic beads were collected and the supernatant was removed. Repeat the operation 1 time.

(3) Cross-linked antibody

Aspirate 217 μL of DMSO into the tube containing DSS, prepare a 10× stock solution (25 mM), and mix by pipetting until DSS is completely dissolved. According to the ratio of 100:1, add DMSO to 10×DSS reagent to prepare DSS with a final concentration of 0.25 mM. Add 4 μL of 0.25 mM DSS, 2.5 μL of 20× coupling buffer and 43.5 μL of ultrapure water to the beads. Incubate with shaking for 30 min, collect the magnetic beads, and save the supernatant. Add 100 μL of elution buffer to the magnetic beads, shake and incubate for 5 min at room temperature, collect the magnetic beads, and discard the supernatant. Add 100 μL of elution buffer to the beads and mix by inversion. Magnetic beads were collected on a magnetic stand and the supernatant was removed. Repeat this step once. Add 200 μL of Piercc Lysis/Wash Solution (pre-chilled) to the beads and mix by inversion. The magnetic beads are enriched and the liquid is removed.

(4) Antigen immunoprecipitation

Aspirate the cell lysate into an EP tube containing antibody-linked magnetic beads, and incubate at 4°C with shaking for 12 h to keep the magnetic beads in suspension. Magnetic beads were collected on a magnetic stand, and the supernatant was saved. Add 500 μL Pierce Lysis/Wash Solution to the EP tube and mix well. The magnetic beads were collected and the supernatant was removed. Repeat 1 time. Add 500 μL of ultrapure water to the EP tube and mix well. The magnetic beads were collected and the supernatant was removed. Add 100 μL of eluate to the EP tube, rotate and incubate for 5 min. The beads are enriched and the eluate is saved. This step can be repeated 1 time. After elution, neutralizing solution was added to the above eluent at a ratio of 1:10 to neutralize the pH of the eluent, and the final product was used for Western blot detection.
