# Supplementary material for: Myostatin Mutation Enhances Bovine Myogenic Differentiation through PI3K/AKT/mTOR Signalling via Removing DNA Methylation of RACK1
Source: Cells. 2022 Dec 23;12(1):59. doi: 10.3390/cells12010059 (PMC9818849; doi:10.3390/cells12010059)
Supplement: Supplementary file 1 [file cells-12-00059-s001.zip › Supplementary Material File S8.pdf]

Hydroxymethylcytosine (5hmC) hydroxylase TET activity assay (ELISA assay)

For our experiments, we used the hydroxymethylcytosine (5hmC) hydroxylase TET activity assay kit (MEIMIAN, Wuhan, China). Cells were washed (PBS pre-cooled), trypsinised for 2 minutes and centrifuged at 1000 × g for 5 min to enrich the pellet. Five concentrations (160 ng/L, 80 ng/L, 40 ng/L, 20 ng/L, 10 ng/L) of standard were obtained by double dilution of the original standard (320 ng/L). Divide the blank wells, standard wells, and test sample wells on the ELISA-coated plate. Add 50 μL of the standard substance to the standard wells; add 40 μL of the sample diluent to the wells of the samples to be tested, and then add 10 μL of the samples to be tested; do not add samples or enzyme labelling reagents to the blank wells and the rest is as described above. Shake gently to mix. The ELISA-coated wells were sealed with a sealing film and heated at 37°C for 30 minutes. Dilute the 30× concentrated wash solution 30:1 with ultrapure water. Discard the sealing foil, spin the liquid dry, fill the wells with wash solution and leave for 30 seconds before discarding the solution. Repeat the procedure 5 times. Add 50 μL of the Enzyme Labelling Reagent to each well (no blank well). The ELISA-coated wells were sealed with a sealing foil and heated at 37°C for 30 min. Discard the sealing membrane, spin dry, fill the wells with wash solution and allow to stand for 30 seconds before discarding the solution. Repeat the procedure 5 times. First add 50 μL of chromogenic reagent A to each well, then the same volume of chromogenic reagent B, shake and mix, and incubate at 37°C for 10 min in the dark. Add 50 μL of the stop solution to each well, and the blue-to-yellow reaction stops. Microplate reader (set wavelength 450 nm) to determine the OD value of each well. The process was completed within 10 minutes of adding the stop solution.
