# Supplementary material for: Myostatin Mutation Enhances Bovine Myogenic Differentiation through PI3K/AKT/mTOR Signalling via Removing DNA Methylation of RACK1
Source: Cells. 2022 Dec 23;12(1):59. doi: 10.3390/cells12010059 (PMC9818849; doi:10.3390/cells12010059)
Supplement: Supplementary file 1 [file cells-12-00059-s001.zip › Supplementary Material File S9.pdf]

Design and synthesis of siRNA

The siRNAs of the gene were designed and synthesized by Guangzhou RiboBio Co., Ltd. (RiboBio, Guangzhou, China). The sequences are detailed in Table 1 and Table 2.

Table 1 Sequences of si-MSTN

Fragment Name	Sequence (5'-3')
si-bta-MSTN_001	TGGCGAAGGACAAATAATA
si-bta-MSTN_002	CACTACATCCTCAAGACTA
si-bta-MSTN_003	GGCTTGATTGTGATGAACA

Table 2 Sequences of si-RACK1

Fragment Name	Sequence (5'-3')
si-bta-RACK1_001	CCAGAGATGAGACCAACTA
si-bta-RACK1_002	GCCATACCAAAGATGTGCT
si-bta-RACK1_003	CTGGGTGTATGCAAGTATA

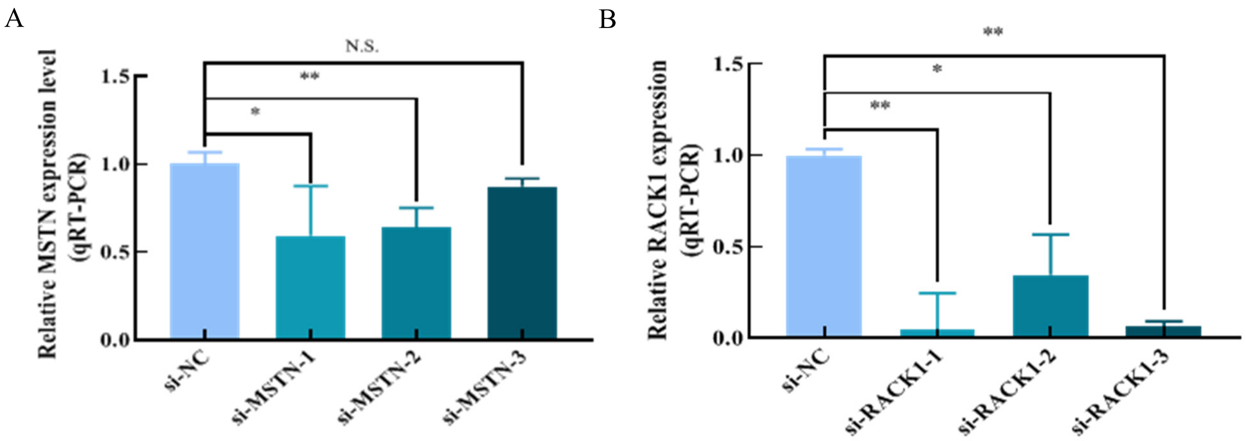

Supplementary Figure. The siRNA interference effect map. A, siRNA inhibits transcription of MSTN. B, siRNA inhibits the transcription of RACK1.

Construction of overexpression vector

The complete sequence of the CDS region of the RACK1 gene (Bos taurus) is from the NCBI website (https://www.ncbi.nlm.nih.gov/) and totals 984 bp. Use

pcDNA3.1(+) plasmid, BamHI and EcoRI restriction sites. PCR amplification and cloning of the full-length CDS region of RACK1 was performed using the bovine genome sequence as a template. Glue recovery to obtain the target sequence. The configuration system of the PCR is shown in Table 3, and the programme settings are shown in Table 4.

Table 3 PCR amplification system

Reagent	Volume（μL）
2*Super Pfx MasterMix	10
Forward Primer	1
Reverse Primer	1
cDNA	2
ddH2O	Up to 20

Table 4 PCR reaction program

Procedure	Temperature	Time	Cycles
Predenaturation	95℃	2min	
Denaturation	95℃	30s	35
Annealing	65℃	30s	
Extension	72℃	1min	
Extension	72℃	2min	

The product concentration of gel recovery was measured. Using the nucleic acid restriction enzymes EcoRI and BamHI, the product and pcDNA3.1(+) plasmid were recovered simultaneously in the digestion gel, and the system was configured according to Table 5. React in a PCR machine at 37°C for 30 minutes. Purify the produc a purification kit, and then ligate with T4 ligase to generate the RACK1 gene expression vector pcDNA3.1(+)-RACK1. Finally, carry out the conversion.

Table 5 Building a carrier configuration system

Reagent	PcDNA3.1(+) volume (μL)
10xFast Digest Buffer	2
EcoRI	1

BamHI	1
DNA	1000ng/concentration
ddH2O	20-X

Transformation process: take 10 μL of the ligation product, add it to 50 μL of competent DH5αcells, and leave it for 25 minutes. Perform a heat shock in a water bath at 42°C for 45 s and then leave in an ice bath for 2 min. Add 500 μL of liquid LB medium without ampicillin to the tube and let it stand at 37° C and 180 rpm for 1 hour. The revival solution was spread on LB solid petri dishes containing ampicillin and left upside down overnight at 37°C in an incubator. The positive bacteria were selected for colony PCR verification. At the same time, the positive bacteria were collected and cultured in liquid LB medium with ampicillin at 37°C and 180 rpm overnight. Finally, the plasmids were extracted for sequencing and comparison.
